# Drug-coated Balloons in the Neurovascular Setting: A Comprehensive, Systematic Review of Current Use and Indications

**DOI:** 10.31083/j.rcm2304128

**Published:** 2022-04-02

**Authors:** Philipp von Gottberg, Alexandru Cimpoca, Christina Wendl, José E. Cohen, Ulrich Speck, Hans Henkes

**Affiliations:** ^1^Neuroradiologische Klinik, Klinikum Stuttgart, 70174 Stuttgart, Germany; ^2^Neuroradiologisches Zentrum, Institut für Röntgendiagnostik, Universitätsklinikum Regensburg, 93053 Regensburg, Germany; ^3^Department of Neurosurgery, Neuroendovascular Center, Hadassah University Hospital, 91120 Jerusalem, Israel; ^4^Klinik für Radiologie, Charité Universitätsmedizin Berlin, 10117 Berlin, Germany; ^5^Medizinische Fakultät, Universität Duisburg-Essen, 45147 Essen, Germany

**Keywords:** drug-coated balloons, percutaneous transluminal angioplasty, neurovascular, neurointerventional, arterial stenosis, carotid artery stenosis, intracranial atherosclerotic stenosis, stroke, paclitaxel

## Abstract

**Background:**

Drug-coated balloons (DCB) are an established tool in the prevention and treatment of coronary and peripheral artery restenosis. The underlying effects of restenosis resemble those in the neurovascular field, yet data on the use of DCB in cervical and intracranial arteries is rare.

**Methods:**

Medline, and international and major national guidelines and recommendations were systematically searched for data addressing the use of DCB in the neurovascular setting.

**Results:**

Of the 1448 relevant records found in Medline, 166 publications were considered for this review.

**Conclusions:**

Data on the use of DCB in the neurovascular setting show a possible benefit over preceding alternatives, such as self-expanding stents, and balloon-mounted or drug-eluting stents. Nonetheless, the role of DCB remains under-researched, and publications remain lacking.

## 1. Introduction

Materials and devices for neurovascular interventions traditionally have a close connection to cardiovascular devices, and many include or are inspired by precursors with a long history of testing. Over the past 15 years, general advances in the fields of neurointervention and neuroimaging, supported by crucial international study results, as well as technical advances in device manufacturing, have led to a surge in neurointerventional procedures and indications. Consequently, the need for more specific interventional instruments and materials increased and stand-alone neurovascular devices, e.g., stent retrievers or certain blood flow modulating implants, have had their breakthrough or had even been newly developed as a result.

But also, devices with long-term proven reliability in the cardiovascular setting were now deployed in the neurovascular setting, particularly angioplasty devices and principles, such as stents and balloons.

However, despite the increase in treatment options for neurovascular atherosclerotic stenosis, neurointerventionalists have not observed the same treatment outcomes as their colleagues in interventional cardiology and have not been able to establish the full range of possible interventional options. Major trials comparing carotid artery stenting (CAS) vs. transluminal endarterectomy (TEA) have been unable to demonstrate the inferiority of the long-established surgical procedure to the newer micro-invasive procedure [1, 2, 3, 4]. Because CAS and TEA have comparable long-term results and different indications, the debate regarding CAS vs. TEA resembles the longstanding debate regarding percutaneous coronary intervention vs. coronary artery bypass grafting [5, 6, 7, 8, 9]. Additionally, the success of coronary artery stenting, particularly small coronary vessel transluminal angioplasty [10, 11, 12], has not been replicated in intracranial atherosclerotic stenosis in major trials: The SAMMPRIS trial has indicated the superiority of aggressive medical treatment to intracranial stent-assisted angioplasty in new arteriosclerotic lesions [13]. In the VISSIT trial, stenting has been found to result in more stroke and death than medical therapy [14]. Since then, research on the interventional treatment of arterial stenosis in the neurovascular setting, particularly the treatment of intracranial arteries, has declined.

However, new techniques and devices have emerged in the meantime, and developments in the field of cardiology have again advanced the development of neurointervention. Thus, drug-coated balloons (DCB) and drug-eluting stents (DES) have become a focus of neurointerventionalists. Although initial, limited data have shown promising results, the potential applications and indications for these devices remain to be elucidated.

This review provides a systematic overview of the literature and guidelines on DCB use and indications in the neurovascular setting.

## 2. Materials and Methods

This systematic review was performed according to the recommendations of the *Cochrane Handbook for Systematic Reviews of Interventions * [15] and the Preferred Reporting Items for Systematic Reviews and Meta-Analyses (PRISMA 2020) statement [16].

Medline was searched through the PubMed interface with no restrictions in language and publication period. Publications suitable for answering the questions of this review were identified on the basis of combinations of the following:

- Article type: no restriction.

- Vascular setting: neurovascular.

- Intervention: DCB, DES.

- Device type: DCB, DES.

- Drugs: drugs applicable to DCB: paclitaxel; “limus-based” drugs, i.e., everolimus, sirolimus, or zotarolimus.

Additionally, current guidelines of the American Heart Association, American Stroke Association, European Society of Cardiology, European Society for Vascular Surgery, and European Stroke Organisation were evaluated for the above items [17, 18].

Among the 1448 records in Medline meeting the search criteria, 241 were selected for screening on the basis of greatest relevance to the covered topics. Among these, 166 publications were identified as eligible for this review.

## 3. Results 

### 3.1 Drug-coated Balloons

#### 3.1.1 History

Since the first percutaneous transluminal coronary angioplasty in 1977, performed by Grüntzig [19], the materials and methods used have evolved from bare-balloon angioplasty to coronary stents, drug-eluting stents and balloons, DCB, and bioresorbable scaffolds [20, 21]. The principle of PTA was rapidly introduced in many vascular areas, in which surgical stenosis treatment was typically used, because it had already been demonstrated to be beneficial in the supraaortic, neurovascular area at the time when PTA was introduced [22, 23].

In 1983, PTA of the extracranial internal carotid artery (ICA) was first described in the literature [24], and 7 years later, suspected cerebral embolization through plaque damaging was addressed to in a groundbreaking way by the first cerebral protection device in a triaxial setup [25]. Despite these developments, major trials, such as SPACE (2004) and Crest (2010), did not demonstrate the superiority of the endovascular method to open surgery [2, 26].

However, it’s a meanwhile frequently performed procedure that’s not only carried out in surgically non-accessible anatomy but is regarded equally to surgical treatment in regard of different risk factors and conditions of both methods. But both methods stimulate neointimal proliferation, a mechanism that is believed to be primarily responsible for restenosis and instant restenosis in the neurovascular setting [27] increasing the risk of new ischemic events and death after successful treatment.

In intracranial arteries, owing to the high risk of complications, endovascular treatment of intracranial atherosclerotic stenosis (ICAS) has rarely been reported after an initial two-case report on PTA of high-grade basilary artery stenosis in 1980 [28]. Stents for intracranial use were first described in emergency or ultima ratio settings of SAH [29, 30] and surgically inaccessible major aneurysms rather than ICAS treatment. Stent-assisted treatment of ICAS was later incorporated into neurovascular interventions [31, 32, 33, 34, 35]. However, concerns regarding the mid- to long-term benefits of endovascular treatment of ICAS arose early [36]. Ultimately, no randomized multicenter trials to date have demonstrated an advantage of stent assisted ICAS treatment over the best medical treatment (SAMMPRIS [13], VISSIT [14]).

A major reason for the devastatingly poor outcomes of stent-treated ICAS is the high incidence of in-stent restenosis, which is again probably caused by neointimal proliferation [37, 38, 39].

The issue of neointimal proliferation and its negative effects on long-term outcomes have also been observed in the field of cardiology and were addressed in the early 2000s already after a retarding effect on neointimal proliferation after short-term exposure to taxane in a porcine model was described [40, 41]. Consequently, the development of DCB began.

DCB are now a part of cardiovascular guidelines [18, 42] and international consensus statements [43, 44], with the caveat that a class effect of DCB should not be presumed, because the results varied among different types of DCB [42, 45].

In the neurovascular setting, the problem of neointimal proliferation leading to in-stent restenosis and restenosis after angioplasty was initially countered by DES, too. However, the effects of DCB observed in non-neurovascular fields led to the introduction of DCB in the neurovascular setting in due course [44].

The primary use of DCB was the prevention and treatment of in-stent restenosis and restenosis of extracranial carotid artery arteriosclerotic stenosis (ECAS) and ICAS, and reported results indicated advantages over bare-balloon angioplasty as well as re-angioplasty, resembling findings in the field of cardiology. This method was pioneered in 2011 and 2012 by Vajda *et al*. [46, 47] with promising results and was only revisited in 2018 [48, 49, 50, 51, 52, 53, 54, 55, 56]. However, despite good results and reviews continuing to advocate for the evaluation of DCB in the treatment and prevention of ECAS and ICAS in in-stent restenosis [57, 58, 59] and restenosis, data addressing the use of DCB and the evaluation of its possible benefits in the neurovascular setting remain rare; consequently, we believe that this field is under-investigated, and publications are lacking.

Table 1 (Ref. [60]) displays commercially available DCB with a CE mark, intended for intracranial use or usable off-label in the neurovascular setting.

**Table 1.** **Commercially available DCB with CE mark, intended for use or usable off-label in the neurovascular field**.

Device name, producer	Drug, dosage (µg/${\mathrm{mm}}^{2}$)	Excipient	Diameter, length (mm)	Guide wire lumen
Agent™, Boston Scientific	PCX, 2	ATBC	2–4, 12–30	0.014´´
AngioSculptX, Cardiva	PCX, 3	Antioxidant, NDGA	2/2.5, 15/10	0.014´´
ELUTAX “3”, AR Baltic Medical	PCX, 2.2	Dextran	1.5–6, 10–40	0.014´´
ELUTAX-SV, Aachen Scientific	PCX, 2.2	Dextran	2-4/10-40	0.014´´
Danubio, Minvasys	PCX, 2.5	BTHC	1.5–4, 10–40	0.014´´
SeQuent® Please NEO, B. Braun	PCX, 3	Iopromide	2–4/10–40	0.014´´
Pantera® Lux™, Biotronik	PCX, 3	BTHC	2–4/10–30	0.014´´
Restore DEB®, Cardionovum	PCX, 3	Shelloic acid	2–4/15–30	0.014´´
Chocolate Touch®, QT Vascular	PCX, 3	N. N., hydrophilic spacer	2.5–6/40–120	0.014´´, 0.018´´
Dior® II, BioStream/BioPath	PCX, 3	Shellac	Stream 2–4/15–30	0.014´´
			BioPath 4–8/20–150	/0.035´´
Essential Pro, iVascular	PCX, 3	N. N.	1.5–4.5/10–40	0.014´´
IN.PACT™ Admiral AV DCB, Medtronic	PCX, 3.5	Urea	4–12, 40–120	0.035´´
Selution SLR™, MedAlliance	SRO, 1	Poly (lactic-co-glycolic acid) (PLGA) + phospholipids [60]	1.5–5/10–40	0.014´´, 0.018´´
Virtue® SAB, Orchestra BioMed	SRO, N. N.	Phospholipid., perforated balloon	N. N.	0.014´´
Magic Touch, Concept Medical	SRO, 1.27	Phospholipid carrier	1.5–12/10–200	0.014´´, 0.018´´, 0.035´´
Protégé/Protégé NC, Wellinq	PCX/3	N. N., hydrophilic coating	2–4.5/10–30	0.014´´
Extender PTCA, Invamed	PCX/1	Iopromide	2–5/10–30	0.014´´

ATBC, acetyl-tributyl citrate; BTHC, n-butyryl tri-n-hexyl citrate; NDGA, nordihydroguaiaretic acid; PCX, paclitaxel; SRO, sirolimus.

#### 3.1.2 Drugs

After testing of several drugs for the prevention of neointimal proliferation in stents and balloons [61, 62, 63, 64, 65], paclitaxel followed by sirolimus have become established in DCB and are currently the main drugs used.

#### 3.1.2.1 Paclitaxel

Paclitaxel is an antimicrotubular drug that stabilizes microtubule polymerization during mitosis, thus inducing cell death [66, 67]. It effectively inhibits coronary neointimal proliferation *in vitro* and *in vivo * [68]. Paclitaxel is highly lipophilic and therefore virtually insoluble in water [69], thus decreasing drug loss during application and drug downstream—i.e., the effect of leaching of paclitaxel distal to the treated stenosis. However, the amount of paclitaxel effectively reaching the vessel wall during DCB angioplasty, is estimated to be only 10% (5–20%):

- 5–30% of loss en route to the destination.

- 40–70% loss during or directly after balloon inflation and deflation through washout.

- 0–30% of the drug may remain unapplied and may be recovered with the balloon after the intervention [70].

This proportion of lost paclitaxel led to suspicions of tissue harm and consequently to investigations of downstream effects, which culminated in a highly regarded, yet methodically and statistically limited meta-analysis connecting the application of paclitaxel-eluting devices to higher mortality in selected studies [71]. The results of this meta-analysis are controversial. Soon after, several contradictory meta-analyses and trials concerning this issue were published [72, 73, 74, 75, 76, 77]. Studies investigating the direct effects of drugs downstream from paclitaxel-coated devices nevertheless did not find relevant harm to (coronary) tissue distal to the point of application in the vessel [70, 78, 79, 80, 81]. Today, the effect of paclitaxel from paclitaxel-containing implants on tissue away from the site of application is considered irrelevant and is no longer directly related to increased mortality [82, 83].

#### 3.1.2.2 Sirolimus

Sirolimus is another name of rapamycin, which is derived from Streptomyces hygroscopius. Its immunosuppressive and antiproliferative effects stem from blocking the cell-cycle specific kinase Target of Rapamycin (TOR) and subsequently halting cell mitosis and inducing cytostasis [84, 85]. The antiproliferative effect of sirolimus discovered in early work [86] has been demonstrated to be highly effective in the treatment of coronary neointimal proliferation and in-stent restenosis and restenosis through DES [87, 88].

Further developments in coating led to the development of sirolimus-coated balloons (SCB), which can be applied in the same manner as paclitaxel-coated balloons (PCB) with sufficient sirolimus transfer to the vessel wall [89]. The advantages of sirolimus over paclitaxel are mainly seen in sirolimus having a broader therapeutic range, larger safety margin, greater success in restenosis treatment than other anti-restenosis drugs, and that it is cytostatic instead of cytotoxic [90, 91, 92].

Several trials have demonstrated that SCB, which emerged in the mid-2010s, are effective in the treatment of coronary neointimal proliferation [93, 94, 95]. The range of commercially available SCB remains narrow, and randomized trials are scant. Currently, no published data are available on the use of SCB in ECAS or ICAS. However, because SCBs are available in the cardiovascular setting, they may one day be used in the neurovascular setting as well.

#### 3.1.3 Different Coating Techniques and Excipients

In the current state of the art of DCB, the coatings contain a drug (e.g., paclitaxel) and a transfer enhancing excipient or spacer, because bare drug-coated devices achieve less transfer of the anti-proliferative drug [70, 96, 97]. The drug on the balloon is dosed in μg/${\mathrm{mm}}^{2}$ balloon surface, and the amount of drug transferred to the vessel wall tissue is directly correlated with the inhibition of neointimal proliferation; however, the magnitude of drug transfer varies significantly [44].

The most common excipients in DCB are derived from modified plant and bacterial substances (e.g., n-butyryl tri-n-hexyl citrate, dextran, nordihydroguaiaretic acid, resveratrol, shellac, and urea), organic plasticizers (e.g., acetyltributylcitrate), magnesium stearate, and the iodine-containing contrast agent iopromide. Urea- and magnesium stearate-containing coatings have been found to strongly inhibit neointimal proliferation in a porcine model [98] and in patients. However, estimating the applied dose *in vivo* (i.e., in atherosclerotic human vessels) remains a challenge. The optimal technique and excipient may be yet unidentified, given that new coating techniques and excipients continue to be developed, and existing ones are being improved.

### 3.2 Use and Current Indications of DCB in the Neurovascular Setting

The primary use of DCB in the neurovascular setting is to treat or prevent vessel stenosis and restenosis, as reflected by the data in the literature.

To date, no official guidelines or recommendations exist regarding the primary use of DCB in the neurovascular setting. However, guidelines consider intracranial angioplasty in emergency situations and bail-out maneuvers [99, 100].

Therefore, interventionalists are dependent on focused, extensive clinical and imaging diagnostics.

#### 3.2.1 Extracranial

3.2.1.1 Internal Carotid Artery

Although *in vivo* experiments with temporal occlusion of the ICA have found that as much as 15% of mean cerebral perfusion is supplied by the ipsilateral external carotid artery [101], to date, no relevant advantage of sole external carotid artery-stenosis treatment—both endovascular and surgical—has been reported [102], apart from case reports focused on rare symptoms and constellations of external carotid artery stenosis. Treatment of the extracranial carotid artery is mainly focused on treatment of the proximal ICA; therefore, this section focuses on ECAS treatment of the ICA.

Use of PTA vs. TEA for the treatment of ECAS has been investigated intensively, yet the clear superiority of either remains unproven. Although TEA appears to raise the risk of a myocardial infarction, the risk of ipsilateral stroke is higher in PTA [103]. However, long-term outcomes may be similar [104]. The advantages and particularly the disadvantages of both treatment types are subject to an ongoing discussion and must be considered for each patient individually.

The use of DCB as an index treatment of external carotid artery stenosis is not common. To our knowledge, no trial or major case study has investigated the effects of using DCB in the PTA treatment of de novo ECAS.

The primary use of DCB is therefore the treatment of ECAS restenosis after TEA and PTA, which is a common issue: in the 2018 ICSS trial, the 5-year cumulative risk of restenosis after TEA and in-stent restenosis after PTA has been reported to be approximately 30% for TEA and 40% for PTA, thus raising the overall risk of ipsilateral stroke in the studied population of 1530 individuals [105].

Challenging ECAS in-stent restenosis (ISRS), the use of DCB was first reported in 2012 in two case series (IN.PACT™ Amphirion, Medtronic, Dublin, Ireland [106] and IN.PACT™ Admiral, Medtronic-Invatec, Dublin, Ireland [48]) describing the application of paclitaxel DCB in an ISRS after standard balloon predilation. At a median follow-up of 15 months, a total of ten treated patients showed not only arrested neointimal hyperplasia in the dilated vessel lumen but also a subsequent decrease in flow peaks on Doppler sonography.

This phenomenon was also reported in a case series of nine patients in 2014 with ISRS refractory to several preceding endovascular maneuvers (DIOR II; Eurocor) [107].

Piccoli *et al*. [108] have published a case series of 18 patients in 2015 with high-grade stenosis after TEA, who were treated with DCB before stent-PTA of the carotid artery. At a mean follow-up of 18 months, no restenosis over 50% of the vessel lumen occurred (IN.PACT™ Pacific balloon, Medtronic Vascular, Dublin, Ireland; Lutonix Balloon, Becton Dickinson, Franklin Lakes, USA).

In a retrospective single-center study, Pohlmann *et al*. [49] (SeQuent® Please, Braun Melsungen, Vascular Systems, Melsungen, Germany; IN.PACT™ Amphirion balloon, Medtronic, Dublin, Ireland) studied nine patients treated with DCB for ISRS (11 procedures in total due to bilateral ISRS in one patient and repeated DCB treatment due to restenosis after 19 months in another patient). In that study, the event-free rate was 100% after the first year and 83% after 5 years of follow-up. No periprocedural strokes or deaths were observed.

The first direct comparison of DCB angioplasty vs. bare balloon angioplasty in ISRS treatment was a single-center prospective registry evaluation, in a study published in 2019 by Tekieli *et al*. [109]. The DCB-group (n = 27) showed a second ISRS after DCB treatment earlier (mean 23 months) than the bare-balloon group (n = 19; mean 26 months). However, the rate of second ISRS was smaller in the DCB-treated group (23%) than in the group with bare balloon treatment (32%). The rate of periprocedural stroke or death was reported to be <2% for the bare-balloon (n = 19), DCB (n = 27) and stent-supported (n = 6) arms and is considered a DCB complication in this review.

Another retrospective single-center study comparing different techniques of endovascular treatment vs. open surgery for ICA restenosis after TEA has not shown significant differences in a 1-year follow-up in terms of overall survival, survival without recurrent restenosis, and survival without reintervention for DCB (n = 18), bare balloon (n = 7), and CAS (n = 9) vs. open surgery (Lutonix, Becton Dickinson) [110]. Moreover, no significant differences among the three endovascular techniques were observed; however, because of the small number of cases, this result has limited validity.

Mihály *et al*. [52] published a comparison of ISRS treatment and re-ISRS treatments in a group of 46 patients with CAS (n = 7) vs. bare balloon (n = 37) vs. DCB (IN.PACT™ Falcon; Medtronic, Dublin, Ireland; Ranger™, Boston Scientific Corp., Marlborough, USA) angioplasty (n = 2) and found no restenosis in the DCB group in a mean follow-up of 30 months. The authors have concluded that DCB is superior to CAS and bare balloon angioplasty in the prevention of ISRS; however, owing to the small number of cases, this result has limited validity.

Table 2 (Ref. [48, 49, 52, 106, 108, 109, 110]) shows the study data in detail.

**Table 2.** **Studies addressing the use of DCB for restenosis treatment of the extracranial carotid artery**.

Author, year	DCB treatment N	Target	Follow-up	Reported outcome	Periprocedural stroke/death
Liistro *et al*. [106], 2012	3	In-stent restenosis	36 months for symptoms, 24 months for stent patency	100% asymptomatic, 100% stent patency	-
Montorsi *et al*. [48], 2012	7	In-stent restenosis	21 ± 19 months, mean 12 months	Significant decrease in in-stent peak systolic velocity	-
Piccoli *et al*. [108], 2015	18	Stenosis after TEA	13–22 months, mean 18 months	Stent patency >50% in 100% of cases	-
Pohlmann *et al*. [49], 2017	9, 11 procedures	In-stent restenosis	5 years	Event-free rate 83%	-
Tekieli *et al*. [109], 2019	27	In-stent restenosis	26 months	Earlier 2nd ISRS after DCB treatment than bare balloon, but at a lower rate in total	2% in total (DCB and bare balloon PTA)
Haupert *et al*. [110], 2020	18	Stenosis after TEA	12 months	Comparable results for DCB vs. CAS vs. bare balloon PTA vs. open surgery/14% restenosis	3% in total (DCB, CAS, and bare balloon PTA)
Mihály *et al*. [52], 2021	2	In-stent restenosis	9–53 months, mean 30 months	No restenosis in DCB-treated patients	Not reported

3.2.1.2 Vertebral Artery

Vertebral artery atherosclerotic stenosis (VAOS) may account for up to 25% of cerebral ischemia cases [111, 112] causing a high rate of mortality due to manifestation in the posterior circulation. The CAVEATS [113], VAST [114], and VIST [115] trials have investigated stent-assisted PTA of the vertebral arteries, and the results have suggested medical treatment as the first line of therapy. However, the ongoing debate about which treatment is better resembles that with ICAS.

Limited data are available regarding the use of DCB as primary treatment for VAOS. Wang *et al*. [116] reported the first case of DCB PTA in a patient with high-grade stenosis originating at a dominant vertebral artery, who did not qualify for stent-assisted PTA. The treatment was uneventful, as was the follow-up at 6 months, and no restenosis was observed on computed tomography angiography after that period.

In 2020, Gruber *et al*. [117] published a retrospective single-center study on 12 patients treated with DCB only for VAOS. No technical failures or instances of restenosis were found after a mean follow-up of 6.1 months, and no periprocedural major adverse events were recorded.

Wang *et al*. [118] have reported a restenosis rate of 10% at 6 months after DCB treatment (Orchid, Acotec Scientific, Beijing, China; Dhalia, Acotec Scientific, Beijing, China) of VAOS in 26 patients. No periprocedural strokes/deaths were reported. The trial was a pilot for a larger, prospective randomized single-center trial comparing VAOS treatment through DCB (PCB, Acotec Scientific, Beijing, China) with distal embolic protection (n = 49) vs. bare metal stenting without embolic protection (n = 46), which was published in 2021 [119]. The technical success rate was comparable in both groups (94% for DBC vs. 96%), and fewer embolic DWI lesions were observed in the DCB group. The restenosis rate was comparable in both groups after the mean follow-up of 12 months (10% DCB vs. 13% bare metal stent). The reported rate of periprocedural major adverse events was 2% in total, with one case in each group.

Table 3 summarizes the results of the described studies.

**Table 3.** **Results of studies investigating DCB as a primary treatment for VAOS**.

Number of studies; *period*	Patients n	Follow-up	Restenosis rate	Periprocedural complication rate
4; 2018, 2020, 2021	88	7.5 months (IQR 6–12)	5% (IQR 0–10)	0.5% (IQR 0–2)

Of note, the role of DCB in the treatment of subclavian artery stenosis is unknown. In the current literature, only a few case reports have addressed this topic, including one case report of successful treatment of ISRS in the subclavian artery [120, 121, 122].

#### 3.2.2 Intracranial

SAMMPRIS (2011/12) and VISSIT (2015) put the use of intracranial bare-metal stents for ICAS treatment on the second line. High-intensity statin and dual- to mono-platelet inhibition [13] has since then been considered first-line. Endovascular treatment is considered in cases of a second stroke in the same vessel territory under medical therapy. However, as devices and strategies have evolved, the idea of “leaving nothing behind” that originated in cardiology has prompted the investigation of the use of DCB as a primary treatment approach for atherosclerotic stenotic vessel disease in the neurovascular setting, too.

In 2018, Gruber *et al*. [50] were the first to publish a comparative retrospective cohort study, investigating the effect of ICAS treatment with a DCB (Neuro Elutax sV, Elutax, Aachen scientific, Aachen, Germany) vs. a bare-metal stent for intracranial stenosis treatment (Wingspan® Stent system, Boston Scientific, Marlborough, USA). In eight patients treated with DCB and 11 patients treated with the stent system, a significantly lower rate of the endpoint combination of ischemic re-event and/or restenosis was observed in the DCB-group (n = 1 DCB vs. n = 7 stent system). The follow-up time was 9.5 months in the DCB group and 10 months in the stent group. No peri- or intraprocedural complications were reported for the DCB-group, but one intraprocedural in-stent thrombosis and one case of temporary hyperperfusion syndrome were reported in the stent group.

In addition, in 2018, Han *et al*. [51] published a retrospective single-center analysis of 30 patients with 31 vessel segments treated for symptomatic high-grade ICAS with DCB (SeQuent® Please, B. Braun Melsungen, Melsungen, Germany). Two patients with periprocedural stroke were reported, and after a mean follow up of 10 ± 3 months, no new ischemic symptoms were observed. Angiographic follow-up revealed one case of asymptomatic restenosis (3%).

Also in 2018, Gruber *et al*. [53] described the primary DCB treatment (SeQuent® Please Neo, B. Braun Melsungen, Melsungen, Germany) of ten patients with high-grade, symptomatic ICAS. They received a median stenosis reduction grade of 28% (IQR 75–80% to IQR 45–53%) without restenosis during the follow-up period of 3 months. No periprocedural adverse events were recorded, and no new ischemic events or deaths were recorded in the follow-up.

Zhang *et al*. [54], in 2020, reported a retrospective study comparing the treatment of high-grade, symptomatic intracranial stenosis with bare-balloon PTA (n = 73) and DCB PTA (SeQuent® Please, B. Braun Melsungen, Melsungen, Germany; n = 42). Both groups achieved favorable outcomes; however, the restenosis rate in the DCB group was significantly lower than that in the bare-balloon group in a 6-month follow-up (DCB restenosis rate 5% vs. 34%). The rate of periprocedural complications was 3% in the DCB-group and 11% in the bare balloon–group.

A Chinese single-center retrospective cohort study published in 2020 [56] described DCB (SeQuent® Please, B. Braun Melsungen, Melsungen, Germany ) for the primary treatment of ICAS with bare balloon predilation. A total of 39 vessel segments in 35 patients were treated, and the angiographic follow-up was 11 ± 4 months. The authors reported a restenosis rate of 8%, with a disproportionately high incidence in intracranial ICA, thus suggesting that the risk of restenosis varies among vascular segments. The reported rate of periprocedural complications was 6% for two major complications.

Remonda *et al*. [123] have described two types of DCB (SeQuent® Please, B. Braun Melsungen, Melsungen, Germany; ELUTAX-SV, Aachen Scientific, Aachen, Germany) for the treatment of high-grade ICAS in a 2021 study with a total of 33 patients and 35 vessel segments treated. In a mean follow-up of 9 months (IQR 3–22 months), restenosis occurred at a rate of 12%. The authors reported a 6% rate of periprocedural complications. The results were not reported according to the balloons used.

In 2020, Yang *et al*. [124] investigated the safety and short-term efficacy of DCB for symptomatic intracranial vertebrobasilar artery stenosis exclusively. A total of 16 treated patients showed good to very good results (0–20% residual stenosis), and no restenosis and new symptoms were observed in the 6-month angiographic and clinical follow-up. The rate of periprocedural complication was 6%.

Table 4 summarizes the results of the described studies.

**Table 4.** **Results of the studies published to date addressing primary treatment of ICAS with DCB**.

Number of studies; *period*	Treated vessel segments N	Follow-up	Restenosis rate	Periprocedural complication rate
7; *2018–2020*	181	7.8 months (IQR 3–11)	6% (IQR 0–13)	3.9% (IQR 0–6.5)

DCB for the treatment of ICAS restenosis and ICAS ISRS is another field of DCB use in the neurovascular setting. However, data on this topic are scarce. The need for this treatment is based on the frequent rate of restenosis of ICAS after balloon and stent treatment. A current review from China on risk factors and the incidence of intracranial ISRS has pooled 5043 patients from 51 studies on the subject and reported a 12–18% rate of relevant restenosis in intracranial stents after the first ICAS treatment at a mean follow-up time of 18 months [125]. Among the patients with ISRS, 29% were symptomatic.

As in cardiology, neointimal proliferation is suspected to be a main factor influencing intracranial ISRS [27], among other factors including thrombus reorganization, elastic return, vascular remodeling, or platelet aggregation [126]. Regarding this pathophysiology, data published in 2011 and 2012 by Vajda *et al*. [46, 47] on the treatment and prevention of intracranial ISRS were promising. In the 2011 study, among 51 patients with intracranial ISRS, 63 maneuvers of restenosis treatment were performed, 41 using a DCB (SeQuent® Please, B. Braun Melsungen, Melsungen, Germany) vs. 20 using a bare balloon. After a mean follow up of 7.5 months with a maximum of 17 months, a restenosis rate of 9% was reported for DCB vs. 50% for bare balloons. The rate of major periprocedural events was 3%.

The data published in 2012 addressed the prevention of ISRS by investigating the effects of using a DCB (SeQuent® Please, B. Braun Melsungen, Melsungen, Germany) before applying a self-expanding stent in symptomatic ICAS. This maneuver, performed in 51 patients, resulted in a restenosis rate of 3% after a mean follow-up of 9 months and a maximum of 16 months. The rate of major adverse events was 5%.

However, both studies have described technical problems with the applied DCB relating to the stiffness of the device, which complicated control and led to technical failure in a minority of cases.

Since then, only Xu *et al*. [55] further investigated the use of DCB (SeQuent® Please, B. Braun Melsungen, Melsungen, Germany) of intracranial ISRS treatment in a study of 11 patients with symptomatic ISRS with DCB. No symptomatic strokes or deaths were reported for the supplied territory of the treated vessel in a 3-month follow-up in this preliminary study.

Table 5 summarizes the results of both studies addressing ISRS treatment with DCB.

**Table 5.** **Results of studies addressing intracranial ISRS treatment with DCB**.

Number of studies; *period*	DCB maneuvers N	Follow-up	Restenosis rate	Periprocedural complication rate
2; 2011, 2020	51	5.3 months (3; 7.5)	4.5% (0; 9)	1.5% (0; 3)

To date, there have been no investigations or recommendations on the use of DCB in cervical or intracranial dissected vessels, nor for occluded vessels (via embolus) or comparable intracranial artery diseases with limited indications for revascularization.

## 4. Discussion

This review addressed the broad range of applications of DCB in the neurovascular setting. The alternatives to DES and DCB in the neurovascular setting were the prior methods of bare-balloon angioplasty, balloon-mounted stents, and self-expandable stents.

Although self-expanding stents, such as the Wingspan® stent system (Stryker), have been discredited by SAMMPRIS and VISSIT, careful patient selection and a focus on findings from medical centers with extensive experience in endovascular treatment have revealed much better results in ICAS treatment than VISSIT/SAMMPRIS [127, 128, 129]. Two large randomized multicenter studies are currently underway and aim to address the safety and effectiveness of self-expanding stent PTA in ICAS treatment. A reassessment of self-expanding stents in ICAS treatment is likely to be imminent. However, these stent systems remain expensive, and, with a two-step deployment technique (after predilation), they may be associated with a higher intrinsic risk of periprocedural complications [130]. The reported rates of periprocedural complication and restenosis are also higher with self-expanding stents than DCB for ICAS treatment, although the comparability is limited. Furthermore, the recent approach of “leaving nothing behind” in stenosis treatment in cardiology is not adhered to with any type of stent treatment. While this alone is not a rationale for the superiority of sole DCB treatment, especially in considering re-stenosis treatment approaches, treatment options for surgically non-accessible vessels of the brain may be reduced after repeated stent treatment.

Balloon-mounted stents use a one-step deployment technique but have not been demonstrated to be superior to self-expanding stents for ICAS treatment in a study of 409 patients with 407 symptomatic ICAS treatments, in terms of complication rates [131]. In a prior evaluation of the same study group focusing on in-hospital complications, a 7% rate of disabling strokes or death was reported in both study arms [132]. Like self-expanding stents, balloon-mounted stents have been reported to be difficult to navigate in the neurovascular setting, which often contains more tortuous vessels than the cardiology and peripheral angioplasty settings [46, 47].

DES for ICAS PTA were initially described in 2005 by Abou-Cheb [133] and Boulos [134]. To date, 15 studies in 480 patients treated for ICAS with DES have been published [135, 136, 137, 138, 139, 140, 141, 142, 143, 144, 145, 146, 147]. Periprocedural complications occurred in an average of 5.3% of cases, and at an imaging follow-up of 4–55 months, the rates of stroke within and after 30 days were reported to be 8% and 2%, respectively, at an ISRS rate of 4% [148]. Despite not being directly comparable, these values surpass the results in SAMMPRIS and VISSIT.

A network meta-analysis of ISRS treatments found the usage of DES and DCB in treatment of coronary ISRS superior to bare-metal-stent-in-stent and bare-balloon-in-stent revascularization in terms of target lesion restenosis reduction [149]. However, many interventionalists view the presence of several metal layers as a limitation of the DES-/bare-metal-stent-in-stent principle, whereas after DCB therapy, further interventional options exist [150].

Nonetheless, DES remain expensive devices, and the long-term effects of their eluted drugs on the brain tissue remain unclear; moreover, they do not adhere to the approach of “leaving nothing behind”. The published data on DES have shown promising results in terms of low restenosis rates, although the periprocedural complication rates may be above those of DCB. However, the recent BASKET SMALL 2 trial [151] in the field of cardiology comparing DCB vs. DES in the treatment of small diameter coronary artery stenosis has indicated the superiority of DCB treatment. This setting may be comparable to the neurovascular setting, thus again favoring DCB.

Bare-balloon angioplasty with consideration of neurovascular features, e.g., submaximal inflation [152], achieved better results than the medical and stent PTA arms in SAMMPRIS in two studies, with 1-year ischemic event-free survival rates of 91% [153] in 2012 and 94.5% [154] in 2016. In a total of 65 patients, the rate of periprocedural complications was also significantly lower than that with stent PTA in SAMMPRIS. Although bare-balloon angioplasty inherits the same procedural steps as DCB angioplasty, the reported rate of periprocedural complications is lower. A reason for this may be that, as in cardiology, the main approach when using a DCB is a DBC-only approach, resulting in careful patient selection and possibly a more careful preparation and application of the procedure and device. Additionally, in the treatment of de novo stenosis without the presence of a stent, the mechanism of late lumen enlargement plays an important additional role. Analogous to the Glagov phenomena, there is a compensatory enlargement of the total vessel cross-section with enlargement of the vessel lumen, which can be seen in paclitaxel-coated DCB when applied in the cardiovascular setting in approximately 2/3 of all cases [155, 156, 157]. If this effect is also active in the neurovascular setting, it may also contribute to a lower rate of complications.

Notably, bare-balloon PTA of ICAS appears to remain a safe and more efficient alternative to bare-metal stent PTA. However, the moderate- to long-term rates of restenosis are unclear. Because bare-balloon PTA devices are inexpensive and relatively easy to navigate in the neurovascular setting, further research on their mid- to long-term benefits is necessary to estimate their value vis-à-vis DCB. The stimulus for neointimal proliferation should be much less than in stent-assisted angioplasty; thus, bare-balloon angioplasty as an initial treatment may achieve comparable results to DCB treatment. However, at least in the prevention and treatment of ISRS, the mid- to long-term results of bare-balloons may be inferior to those of DCB-treatment as bare-balloons do not include protection from neointimal proliferation.

Also, a major concern of the intracranial use of DCB is embolism through particle coating downstream, leading to embolic stroke. Bare-balloon catheters do not have this issue, raising the question of whether DCB-angioplasty may carry a higher risk of stroke. A study by Colleran *et al*. [81] with n = 343 patients checked for signs of cardiac infarction following bare-balloon angioplasty (n = 116), DCB angioplasty (n = 115) and DES-angioplasty (n = 112). It showed no difference in the three study arms in terms of periprocedural or early postprocedural signs of cardiac infarction [81]. There are no data comparing the rate of embolic infarction following intracranial bare-balloon angioplasty vs. DCB angioplasty; however, when comparing the mass of debris collected distal to the saphenous vein, as well as cardiac and carotid arteries after angioplasty [158, 159], the amount of collected debris with a mean of 16 ${\mathrm{mm}}^{3}$ is far above the volume/amount of coated material on a DCB in total, which can be estimated to be approximately 1 ${\mathrm{mm}}^{3}$ or 9 mg, respectively [70]. The burden from plaque debris would therefore play a major role in downstream infarction, despite not being connected to the kind of balloon.

When considering the pharmacological effect of Paclitaxel, there is concern for brain cells being exposed to this toxin. Currently, there are no data on the toxicity of Paclitaxel on neurons after ICAS treatment through DCB. The toxic effect as well as the dose applied to neurons downstream after DCB can only be estimated: The whole dose on one DCB suitable for intracranial use is approximately 9 mg of Paclitaxel. Systemic doses used in oncology are approximately 300 mg while a single systemic Paclitaxel dose capable of causing peripheral neuropathy may be 70 ${\mathrm{mg/m}}^{2}$ or higher [160]. An estimated 80–95% of the DCB Paclitaxel is not being transferred to the vessel wall. Thus, in in a worst-case scenario, approximately 8.6 mg of Paclitaxel would be available for downstream effects. There have been no reports of adverse events that could be related to the pharmacological effect of Paclitaxel on neurons, however, and they may be clinically inapparent, negligible or simply covered by overlaying and dominating symptoms following debris embolism.

In the treatment of extracranial arteries of the neurovascular setting, repeated CAS and bare-balloon-PTA are common. In a 2021 review [161] of results from 35 studies with a total of 1374 cases of ECAS ISRS, the reported rate of in-hospital adverse events was 1.1% for CAS as well as for bare balloon PTA, whereas the long-term rate of stroke and TIA was significantly higher with bare-balloon PTA than repeated CAS (6% vs. 2%). The mean rate of restenosis was 28% with bare-balloon PTA and 8% with CAS, both of which exceeded the published rates of restenosis in DCB.

In VAOS treatment, the use of bare-metal stents and DES has rarely been reported in the literature, yet endovascular treatment remains the only alternative to best medical treatment, because open surgery has been abandoned, owing to anatomic difficulties. In reviews and meta-analyses, the rate of DES restenosis has been found to be 12%, in contrast to 29% with bare-metal stents [162, 163], after a mean follow-up of 8 months. The periprocedural complication rate was 2% for DES and 5% for bare-metal stents, with a total death rate at the end of follow-up of 7% with DES and 10% with bare-metal stents.

Another retrospective single-center study [164] of 32 DES in 30 patients in 2019 has reported a major periprocedural complication rate of 6.3% (n = 2) and a restenosis rate of 8% (n = 4) after a mean imaging follow-up of 8.8 months. DCB, in comparison, may yield better results in terms of mid-term patency and the periprocedural complication rate.

Table 6 (Ref. [104, 147, 148, 154, 161, 162, 163, 165, 166]) compares the data on alternative devices and techniques to DCB.

**Table 6.** **Data published in the described studies and trials on different devices for the treatment of intra- and extracranial atherosclerotic stenotic disease**.

Periprocedural complication rate %	DCB	DES	Bare-metal stent	Bare-balloon PTA	Surgery
Extracranial carotid artery	0.7	n/a	5.3 [104]	6 [154]	5.1 [163]
Extracranial vertebral artery	0.5	4	5	1.1	n/a
Intracranial	3.9 in de novo	5.3	9, 6.2 without SAMMPRIS	n/a	n/a
	1.5 in restenosis				
Restenosis % in mean follow-up months	DCB	DES	Bare-metal stent	Bare-balloon PTA	Surgery
Extracranial carotid artery	9% in 26 months	n/a	8% in 17.4 months	28% in 17.4 months	12% in 20 months [165, 166]
Extracranial vertebral artery	5% in 7.5 months	10% in 8.4 months [161, 162, 163]	29% in 8.8 months [161, 162]	n/a	n/a
Intracranial	6% in 7.8 months de novo	4.8% in 27 months [147, 148]	21% in 12 months, 16% in 12 months without VISSIT	n/a	n/a
	4.5% in 5.3 months restenosis				

## 5. Conclusions

Based on the pathophysiological and technical background of atherosclerotic disease in the neurovascular setting, the issues currently faced by neurointerventionalists resemble those encountered by cardiologists years ago.

Whereas restenosis treatment and prevention have been successfully counteracted with DES and DCB in cardiology, in the neurovascular field, since the publication of data from large randomized multicenter trials more than 10 years ago, further research on endovascular techniques and devices for the treatment of stenotic/restenotic disease as well as possible benefits of DES and DCB has declined sharply or has not yet begun.

A comparison of the scant data in the literature regarding the use of DCB in the neurovascular setting suggests possible benefits but also highlights the continued lack of research and publications in this area.

To attempt a reconsideration of the treatment of extra- and intracranial stenosis after CREST, SAMMPRIS and VISSIT, we therefore strongly advocate intensified research on DCBs neurovascular use.

## Abbreviations

CAS, carotid artery stenting; DCB, drug-coated balloon; DES, drug-eluting stent; ECAS, extracranial carotid artery stenosis; ICAS, intracranial artery stenosis; IQR, interquartile range; ISRS, instant restenosis; PCB, paclitaxel-coated balloons; PTA, percutaneous transluminal angioplasty; SCB, sirolimus-coated balloons; TEA, transluminal endarterectomy; VAOS, vertebral artery atherosclerotic stenosis.

## References

[1] Bonati LH, Dobson J, Featherstone RL, Ederle J, van der Worp HB, de Borst GJ (2015). Long-term outcomes after stenting versus endarterectomy for treatment of symptomatic carotid stenosis: the International Carotid Stenting Study (ICSS) randomised trial. *The Lancet*.

[2] Brott TG, Hobson RW, Howard G, Roubin GS, Clark WM, Brooks W (2010). Stenting versus endarterectomy for treatment of carotid-artery stenosis. *The New England Journal of Medicine*.

[3] Halliday A, Bulbulia R, Bonati LH, Chester J, Cradduck-Bamford A, Peto R (2021). Second asymptomatic carotid surgery trial (ACST-2): a randomised comparison of carotid artery stenting versus carotid endarterectomy. *The Lancet*.

[4] Featherstone RL, Dobson J, Ederle J, Doig D, Bonati LH, Morris S (2016). Carotid artery stenting compared with endarterectomy in patients with symptomatic carotid stenosis (International Carotid Stenting Study): a randomised controlled trial with cost-effectiveness analysis. *Health Technology Assessment*.

[5] Spadaccio C, Benedetto U (2018). Coronary artery bypass grafting (CABG) vs. percutaneous coronary intervention (PCI) in the treatment of multivessel coronary disease: quo vadis? -a review of the evidences on coronary artery disease. *Annals of Cardiothoracic Surgery*.

[6] Serruys PW, Morice M, Kappetein AP, Colombo A, Holmes DR, Mack MJ (2009). Percutaneous Coronary Intervention versus Coronary-Artery Bypass Grafting for Severe Coronary Artery Disease. *New England Journal of Medicine*.

[7] Daoulah A, Alasmari A, Hersi AS, Alshehri M, Garni TA, Abuelatta R (2021). Percutaneous Coronary Intervention Vs Coronary Artery Bypass Surgery for Unprotected Left Main Coronary Disease: G-LM Registry. *Current Problems in Cardiology*.

[8] Yang BG, Yu XP, Chen F, Lyu SZ, Li Q, He JQ (2017). Comparison on the long-term outcomes post percutaneous coronary intervention or coronary artery bypass grafting for bifurcation lesions in unprotected left main coronary artery. *Chinese Journal of Cardiology*.

[9] Stachon P, Kaier K, Hehn P, Peikert A, Wolf D, Oettinger V (2021). Coronary artery bypass grafting versus stent implantation in patients with chronic coronary syndrome and left main disease: insights from a register throughout Germany. *Clinical Research in Cardiology*.

[10] Park SW, Lee CW, Hong MK, Kim JJ, Cho GY, Nah DY (2000). Randomized comparison of coronary stenting with optimal balloon angioplasty for treatment of lesions in small coronary arteries. *European Heart Journal*.

[11] Savage MP, Fischman DL, Rake R, Leon MB, Schatz RA, Penn I (1998). Efficacy of coronary stenting versus balloon angioplasty in small coronary arteries. Stent Restenosis Study (STRESS) Investigators. *Journal of the American College of Cardiology*.

[12] Hanekamp C, Koolen J, Bonnier H, Oldroyd K, de Boer M, Heublein B (2004). Randomized comparison of balloon angioplasty versus silicon carbon-coated stent implantation for de novo lesions in small coronary arteries. *The American Journal of Cardiology*.

[13] Chimowitz MI, Lynn MJ, Derdeyn CP, Turan TN, Fiorella D, Lane BF (2011). Stenting versus aggressive medical therapy for intracranial arterial stenosis. *The New England Journal of Medicine*.

[14] Zaidat OO, Fitzsimmons B, Woodward BK, Wang Z, Killer-Oberpfalzer M, Wakhloo A (2015). Effect of a balloon-expandable intracranial stent vs medical therapy on risk of stroke in patients with symptomatic intracranial stenosis: the VISSIT randomized clinical trial. *The Journal of the American Medical Association*.

[15] Higgins JPT, Thomas J, Chandler J, Cumpston M, Li T, Page MJ (2020). Cochrane handbook for systematic reviews of interventions. 2nd edn.

[16] Page MJ, McKenzie JE, Bossuyt PM, Boutron I, Hoffmann TC, Mulrow CD (2021). The PRISMA 2020 statement: An updated guideline for reporting systematic reviews. *Journal of Clinical Epidemiology*.

[17] Kleindorfer DO, Towfighi A, Chaturvedi S, Cockroft KM, Gutierrez J, Lombardi-Hill D (2021). 2021 Guideline for the Prevention of Stroke in Patients with Stroke and Transient Ischemic Attack: a Guideline from the American Heart Association/American Stroke Association. *Stroke*.

[18] Aboyans V, Ricco JB, Bartelink MLEL, Björck M, Brodmann M, Cohnert T (2018). 2017 ESC Guidelines on the Diagnosis and Treatment of Peripheral Arterial Diseases, in collaboration with the European Society for Vascular Surgery (ESVS): Document covering atherosclerotic disease of extracranial carotid and vertebral, mesenteric, renal, upper and lower extremity arteriesEndorsed by: the European Stroke Organization (ESO)The Task Force for the Diagnosis and Treatment of Peripheral Arterial Diseases of the European Society of Cardiology (ESC) and of the European Society for Vascular Surgery (ESVS). *European Heart Journal*.

[19] Gruntzig A (1978). Transluminal dilatation of coronary-artery stenosis. *The Lancet*.

[20] Meier B, Bachmann D, Lüscher T (2003). 25 years of coronary angioplasty: almost a fairy tale. *The Lancet*.

[21] The Lancet (2017). 40 years of percutaneous coronary intervention: where next. *Lancet*.

[22] (1998). Randomised trial of endarterectomy for recently symptomatic carotid stenosis: final results of the MRC European Carotid Surgery Trial (ECST). *Lancet*.

[23] Barnett HJ, Taylor DW, Eliasziw M, Fox AJ, Ferguson GG, Haynes RB (1998). Benefit of carotid endarterectomy in patients with symptomatic moderate or severe stenosis. North American Symptomatic Carotid Endarterectomy Trial Collaborators. *The New England Journal of Medicine*.

[24] Bockenheimer SA, Mathias K (1983). Percutaneous transluminal angioplasty in arteriosclerotic internal carotid artery stenosis. *AJNR. American Journal of Neuroradiology*.

[25] Theron J, Courtheoux P, Alachkar F, Bouvard G, Maiza D (1990). New triple coaxial catheter system for carotid angioplasty with cerebral protection. *AJNR. American Journal of Neuroradiology*.

[26] Ringleb PA, Kunze A, Allenberg JR, Hennerici MG, Jansen O, Maurer PC (2004). The Stent-Supported Percutaneous Angioplasty of the Carotid Artery vs. Endarterectomy Trial. *Cerebrovascular Diseases*.

[27] Papafaklis MI, Chatzizisis YS, Naka KK, Giannoglou GD, Michalis LK (2012). Drug-eluting stent restenosis: effect of drug type, release kinetics, hemodynamics and coating strategy. *Pharmacology & Therapeutics*.

[28] Sundt TM, Smith HC, Campbell JK, Vlietstra RE, Cucchiara RF, Stanson AW (1980). Transluminal angioplasty for basilar artery stenosis. *Mayo Clinic Proceedings*.

[29] Higashida RT, Smith W, Gress D, Urwin R, Dowd CF, Balousek PA (1997). Intravascular stent and endovascular coil placement for a ruptured fusiform aneurysm of the basilar artery. Case report and review of the literature. *Journal of Neurosurgery*.

[30] Sekhon LH, Morgan MK, Sorby W, Grinnell V (1998). Combined endovascular stent implantation and endosaccular coil placement for the treatment of a wide-necked vertebral artery aneurysm: technical case report. *Neurosurgery*.

[31] Al-Mubarak N, Gomez CR, Vitek JJ, Roubin GS (1998). Stenting of symptomatic stenosis of the intracranial internal carotid artery. *AJNR. American Journal of Neuroradiology*.

[32] Phatouros CC, Higashida RT, Malek AM, Smith WS, Mully TW, DeArmond SJ (1999). Endovascular stenting of an acutely thrombosed basilar artery: technical case report and review of the literature. *Neurosurgery*.

[33] Mori T, Kazita K, Chokyu K, Mori K (1999). Stenting treatment for intra- and extra-cranial atherosclerotic diseases. *Interventional Neuroradiology*.

[34] Horowitz MB, Pride GL, Graybeal DF, Purdy PD (1999). Percutaneous transluminal angioplasty and stenting of midbasilar stenoses: three technical case reports and literature review. *Neurosurgery*.

[35] Morris PP, Martin EM, Regan J, Braden G (1999). Intracranial deployment of coronary stents for symptomatic atherosclerotic disease. *AJNR. American Journal of Neuroradiology*.

[36] Gupta R, Schumacher HC, Mangla S, Meyers PM, Duong H, Khandji AG (2003). Urgent endovascular revascularization for symptomatic intracranial atherosclerotic stenosis. *Neurology*.

[37] Cremers B, Toner JL, Schwartz LB, von Oepen R, Speck U, Kaufels N (2012). Inhibition of neointimal hyperplasia with a novel zotarolimus coated balloon catheter. *Clinical Research in Cardiology*.

[38] Jin M, Fu X, Wei Y, Du B, Xu X, Jiang W (2013). Higher risk of recurrent ischemic events in patients with intracranial in-stent restenosis. *Stroke*.

[39] Derdeyn CP, Fiorella D, Lynn MJ, Turan TN, Cotsonis GA, Lane BF (2017). Nonprocedural Symptomatic Infarction and in-Stent Restenosis after Intracranial Angioplasty and Stenting in the SAMMPRIS Trial (Stenting and Aggressive Medical Management for the Prevention of Recurrent Stroke in Intracranial Stenosis). *Stroke*.

[40] Scheller B, Speck U, Romeike B, Schmitt A, Sovak M, Böhm M (2003). Contrast media as carriers for local drug delivery. Successful inhibition of neointimal proliferation in the porcine coronary stent model. *European Heart Journal*.

[41] Scheller B, Speck U, Schmitt A, Böhm M, Nickenig G (2003). Addition of paclitaxel to contrast media prevents restenosis after coronary stent implantation. *Journal of the American College of Cardiology*.

[42] Neumann F, Sousa-Uva M, Ahlsson A, Alfonso F, Banning AP, Benedetto U (2019). 2018 ESC/EACTS Guidelines on myocardial revascularization. *EuroIntervention*.

[43] Jeger RV, Eccleshall S, Wan Ahmad WA, Ge J, Poerner TC, Shin E (2020). Drug-Coated Balloons for Coronary Artery Disease: Third Report of the International DCB Consensus Group. *JACC: Cardiovascular Interventions*.

[44] Cortese B, Granada JF, Scheller B, Schneider PA, Tepe G, Scheinert D (2016). Drug-coated balloon treatment for lower extremity vascular disease intervention: an international positioning document. *European Heart Journal*.

[45] Gongora CA, Shibuya M, Wessler JD, McGregor J, Tellez A, Cheng Y (2015). Impact of Paclitaxel Dose on Tissue Pharmacokinetics and Vascular Healing: a Comparative Drug-Coated Balloon Study in the Familial Hypercholesterolemic Swine Model of Superficial Femoral in-Stent Restenosis. *JACC: Cardiovascular Interventions*.

[46] Vajda Z, Güthe T, Perez MA, Heuschmid A, Schmid E, Bäzner H (2011). Neurovascular in-stent stenoses: treatment with conventional and drug-eluting balloons. *AJNR. American Journal of Neuroradiology*.

[47] Vajda Z, Güthe T, Perez MA, Kurre W, Schmid E, Bäzner H (2013). Prevention of intracranial in-stent restenoses: predilatation with a drug eluting balloon, followed by the deployment of a self-expanding stent. *Cardiovascular and Interventional Radiology*.

[48] Montorsi P, Galli S, Ravagnani PM, Trabattoni D, Fabbiocchi F, Lualdi A (2012). Drug-eluting balloon for treatment of in-stent restenosis after carotid artery stenting: preliminary report. *Journal of Endovascular Therapy*.

[49] Pohlmann C, Höltje J, Zeile M, Bonk F, Urban PP, Brüning R (2018). Recurrent stenosis following carotid artery stenting treated with a drug-eluting balloon: a single-center retrospective analysis. *Neuroradiology*.

[50] Gruber P, Garcia-Esperon C, Berberat J, Kahles T, Hlavica M, Anon J (2018). Neuro Elutax SV drug-eluting balloon versus Wingspan stent system in symptomatic intracranial high-grade stenosis: a single-center experience. *Journal of Neurointerventional Surgery*.

[51] Han J, Zhang J, Zhang X, Zhang J, Song Y, Zhao W (2019). Drug-coated balloons for the treatment of symptomatic intracranial atherosclerosis: initial experience and follow-up outcome. *Journal of Neurointerventional Surgery*.

[52] Mihály Z, Vértes M, Entz L, Dósa E (2021). Treatment and Predictors of Recurrent Internal Carotid Artery in-Stent Restenosis. *Vascular and Endovascular Surgery*.

[53] Gruber P, Braun C, Kahles T, Hlavica M, Anon J, Diepers M (2019). Percutaneous transluminal angioplasty using the novel drug-coated balloon catheter SeQuent Please NEO for the treatment of symptomatic intracranial severe stenosis: feasibility and safety study. *Journal of Neurointerventional Surgery*.

[54] Zhang J, Zhang X, Zhang J, Song Y, Zheng M, Sun L (2020). Drug-Coated Balloon Dilation Compared with Conventional Stenting Angioplasty for Intracranial Atherosclerotic Disease. *Neurosurgery*.

[55] Xu H, Fu X, Yuan Y, Quan T, Wang Z, Han K (2020). Feasibility and Safety of Paclitaxel-Coated Balloon Angioplasty for the Treatment of Intracranial Symptomatic In-Stent Restenosis. *Frontiers in Neurology*.

[56] Wang AY, Chang C, Chen C, Wu Y, Lin C, Chen C (2021). Leave nothing behind: Treatment of Intracranial Atherosclerotic Disease with Drug-Coated Balloon Angioplasty. *Clinical Neuroradiology*.

[57] Padalia A, Sambursky JA, Skinner C, Moureiden M (2018). Percutaneous Transluminal Angioplasty with Stent Placement versus Best Medical Therapy alone in Symptomatic Intracranial Arterial Stenosis: a Best Evidence Review. *Cureus*.

[58] Gruber P, Singh S, Andereggen L, Berberat J, Remonda L (2021). Drug-Coated Balloons for the Treatment of Symptomatic Intracranial High-Grade Stenosis: A Review of the Current Rationale. *Frontiers in Neurology*.

[59] Bhatia K, Akhtar IN, Akinci Y, Liaqat J, Siddiq F, Gomez CR (2020). Drug‐Eluting Balloon Angioplasty for in‐Stent Restenosis Following Carotid Artery Stent Placement. *Journal of Neuroimaging*.

[60] Böhme T, Noory E, Beschorner U, Macharzina R, Zeller T (2021). The SELUTION SLR™ drug-eluting balloon system for the treatment of symptomatic femoropopliteal lesions. *Future Cardiology*.

[61] Claessen BE, Henriques JPS, Dangas GD (2010). Clinical studies with sirolimus, zotarolimus, everolimus, and biolimus a9 drug-eluting stent systems. *Current Pharmaceutical Design*.

[62] Speck U, Scheller B, Rutsch W, Laule M, Stangl V (2011). Local drug delivery – the early Berlin experience: single drug administration versus sustained release. *EuroIntervention*.

[63] Lee MG, Jeong MH, Ahn Y, Cho JG, Park JC, Kang JC (2011). Comparison of paclitaxel-, sirolimus-, and zotarolimus-eluting stents in patients with acute ST-segment elevation myocardial infarction and metabolic syndrome. *Circulation Journal*.

[64] Park KW, Lim W, Kim J, Kang S, Seo J, Song YB (2013). Comparison between zotarolimus-eluting stents and first generation drug-eluting stents in the treatment of patients with acute ST-segment elevation myocardial infarction. *International Journal of Cardiology*.

[65] Sun S, Zhang Q, Wang Q, Wu Q, Xu G, Chang P (2018). Local delivery of thalidomide to inhibit neointima formation in rat model with artery injury. *Pathology, Research and Practice*.

[66] Parness J, Horwitz SB (1981). Taxol binds to polymerized tubulin in vitro. *The Journal of Cell Biology*.

[67] Schiff PB, Horwitz SB (1981). Taxol assembles tubulin in the absence of exogenous guanosine 5’-triphosphate or microtubule-associated proteins. *Biochemistry*.

[68] Axel DI, Kunert W, Göggelmann C, Oberhoff M, Herdeg C, Küttner A (1997). Paclitaxel inhibits arterial smooth muscle cell proliferation and migration in vitro and *in vivo* using local drug delivery. *Circulation*.

[69] Singla AK, Garg A, Aggarwal D (2002). Paclitaxel and its formulations. *International Journal of Pharmaceutics*.

[70] Speck U, Stolzenburg N, Peters D, Scheller B (2016). How does a drug-coated balloon work? Overview of coating techniques and their impact. *The Journal of Cardiovascular Surgery*.

[71] Katsanos K, Spiliopoulos S, Kitrou P, Krokidis M, Karnabatidis D (2018). Risk of Death Following Application of Paclitaxel‐Coated Balloons and Stents in the Femoropopliteal Artery of the Leg: a Systematic Review and Meta‐Analysis of Randomized Controlled Trials. *Journal of the American Heart Association*.

[72] Takahara M, Iida O, Soga Y, Kawasaki D, Fujihara M (2019). Mortality risk of femoropopliteal paclitaxel-coated devices remains inconclusive. *Cardiovascular Intervention and Therapeutics*.

[73] Bonassi S (2019). Letter by Bonassi Regarding Article, “Risk of Death Following Application of Paclitaxel‐Coated Balloons and Stents in the Femoropopliteal Artery of the Leg: a Systematic Review and Meta‐Analysis of Randomized Controlled Trials”. *Journal of the American Heart Association*.

[74] Yerasi C, Case BC, Forrestal BJ, Kolm P, Dan K, Torguson R (2020). Risk of Mortality with Paclitaxel Drug-Coated Balloon in De Novo Coronary Artery Disease. *Cardiovascular Revascularization Medicine*.

[75] Schneider PA, Laird JR, Doros G, Gao Q, Ansel G, Brodmann M (2019). Mortality not Correlated with Paclitaxel Exposure: an Independent Patient-Level Meta-analysis of a Drug-Coated Balloon. *Journal of the American College of Cardiology*.

[76] Scheller B, Vukadinovic D, Jeger R, Rissanen TT, Scholz SS, Byrne R (2020). Survival after Coronary Revascularization with Paclitaxel-Coated Balloons. *Journal of the American College of Cardiology*.

[77] Kumins NH, King AH, Ambani RN, Thomas JP, Bose S, Shishehbor MH (2020). Paclitaxel-coated peripheral artery devices are not associated with increased mortality. *Journal of Vascular Surgery*.

[78] Thompson CA, Huibregtse B, Poff B, Wilson GJ (2009). Time dependent vascular and myocardial responses of a second generation, small vessel, paclitaxel-eluting stent platform. *Catheterization and Cardiovascular Interventions*.

[79] Cremers B, Schmitmeier S, Clever YP, Gershony G, Speck U, Scheller B (2014). Inhibition of neo-intimal hyperplasia in porcine coronary arteries utilizing a novel paclitaxel-coated scoring balloon catheter. *Catheterization and Cardiovascular Interventions*.

[80] Torii S, Yahagi K, Mori H, Harari E, Romero ME, Kolodgie FD (2018). Biologic Drug Effect and Particulate Embolization of Drug-Eluting Stents versus Drug-Coated Balloons in Healthy Swine Femoropopliteal Arteries. *Journal of Vascular and Interventional Radiology*.

[81] Colleran R, Harada Y, Kufner S, Giacoppo D, Joner M, Cassese S (2017). Changes in high-sensitivity troponin after drug-coated balloon angioplasty for drug-eluting stent restenosis. *EuroIntervention*.

[82] Secemsky EA, Kundi H, Weinberg I, Jaff MR, Krawisz A, Parikh SA (2019). Association of Survival with Femoropopliteal Artery Revascularization with Drug-Coated Devices. *JAMA Cardiology*.

[83] Freisinger E, Koeppe J, Gerss J, Goerlich D, Malyar NM, Marschall U (2020). Mortality after use of paclitaxel-based devices in peripheral arteries: a real-world safety analysis. *European Heart Journal*.

[84] Marx SO, Marks AR (2001). Bench to bedside: the development of rapamycin and its application to stent restenosis. *Circulation*.

[85] Sehgal SN (2003). Sirolimus: its discovery, biological properties, and mechanism of action. *Transplantation Proceedings*.

[86] Carter AJ, Aggarwal M, Kopia GA, Tio F, Tsao PS, Kolata R (2004). Long-term effects of polymer-based, slow-release, sirolimus-eluting stents in a porcine coronary model. *Cardiovascular Research*.

[87] Piscione F, Piccolo R, Cassese S, Galasso G, Chiariello M (2009). Clinical impact of sirolimus-eluting stent in ST-segment elevation myocardial infarction: a meta-analysis of randomized clinical trials. *Catheterization and Cardiovascular Interventions*.

[88] Piccolo R, Cassese S, Galasso G, Niglio T, De Rosa R, De Biase C (2012). Long-term clinical outcomes following sirolimus-eluting stent implantation in patients with acute myocardial infarction. a meta-analysis of randomized trials. *Clinical Research in Cardiology*.

[89] Clever YP, Peters D, Calisse J, Bettink S, Berg MC, Sperling C (2016). Novel Sirolimus-Coated Balloon Catheter: *in vivo* Evaluation in a Porcine Coronary Model. *Circulation. Cardiovascular Interventions*.

[90] Wessely R, Kastrati A, Mehilli J, Dibra A, Pache J, Schömig A (2007). Randomized trial of rapamycin- and paclitaxel-eluting stents with identical biodegradable polymeric coating and design. *European Heart Journal*.

[91] Wessely R, Blaich B, Belaiba RS, Merl S, Görlach A, Kastrati A (2007). Comparative characterization of cellular and molecular anti-restenotic profiles of paclitaxel and sirolimus. Implications for local drug delivery. *Thrombosis and Haemostasis*.

[92] Dangas GD, Serruys PW, Kereiakes DJ, Hermiller J, Rizvi A, Newman W (2013). Meta-analysis of everolimus-eluting versus paclitaxel-eluting stents in coronary artery disease: final 3-year results of the SPIRIT clinical trials program (Clinical Evaluation of the Xience V Everolimus Eluting Coronary Stent System in the Treatment of Patients with De Novo Native Coronary Artery Lesions). *JACC: Cardiovascular Interventions*.

[93] Verheye S, Vrolix M, Kumsars I, Erglis A, Sondore D, Agostoni P (2017). The SABRE Trial (Sirolimus Angioplasty Balloon for Coronary in-Stent Restenosis): Angiographic Results and 1-Year Clinical Outcomes. *JACC: Cardiovascular Interventions*.

[94] Ali RM, Abdul Kader MASK, Wan Ahmad WA, Ong TK, Liew HB, Omar A (2019). Treatment of Coronary Drug-Eluting Stent Restenosis by a Sirolimus- or Paclitaxel-Coated Balloon. *JACC: Cardiovascular Interventions*.

[95] Cortese B, Caiazzo G, Di Palma G, De Rosa S (2021). Comparison between Sirolimus- and Paclitaxel-Coated Balloon for Revascularization of Coronary Arteries: the SIRPAC (SIRolimus-PAClitaxel) Study. *Cardiovascular Revascularization Medicine*.

[96] Banning AP, Lim CCS (2010). Drug-eluting balloons: what is their place on the interventionalist’s shelf. *Heart*.

[97] Liistro F, Grotti S, Porto I, Angioli P, Ricci L, Ducci K (2013). Drug-eluting balloon in peripheral intervention for the superficial femoral artery: the DEBATE-SFA randomized trial (drug eluting balloon in peripheral intervention for the superficial femoral artery). *JACC: Cardiovascular Interventions*.

[98] Bettink S, Löchel M, Peters D, Haider W, Speck U, Scheller B (2021). Efficacy and safety of a magnesium stearate paclitaxel coated balloon catheter in the porcine coronary model. *International Journal of Cardiology*.

[99] Kernan WN, Ovbiagele B, Black HR, Bravata DM, Chimowitz MI, Ezekowitz MD (2014). Guidelines for the prevention of stroke in patients with stroke and transient ischemic attack: a guideline for healthcare professionals from the American Heart Association/American Stroke Association. *Stroke*.

[100] Schirmer JH, Aries PM, Balzer K, Berlit P, Bley TA, Buttgereit F (2020). S2k-Leitlinie: Management der Großgefäßvaskulitiden. *Zeitschrift FüR Rheumatologie*.

[101] Fearn SJ, Picton AJ, Mortimer AJ, Parry AD, McCollum CN (2000). The contribution of the external carotid artery to cerebral perfusion in carotid disease. *Journal of Vascular Surgery*.

[102] de Borst GJ, Moll FL (2012). Commentary: selective treatment of external carotid artery stenosis. *Journal of Endovascular Therapy*.

[103] Bonati LH, Kakkos S, Berkefeld J, de Borst GJ, Bulbulia R, Halliday A (2021). European Stroke Organisation guideline on endarterectomy and stenting for carotid artery stenosis. *European Stroke Journal*.

[104] Matsumura JS, Hanlon BM, Rosenfield K, Voeks JH, Howard G, Roubin GS (2021). Treatment of carotid stenosis in asymptomatic, non-octogenarian, standard risk patients with stenting versus endarterectomy trials. *Journal of Vascular Surgery*.

[105] Bonati LH, Gregson J, Dobson J, McCabe DJH, Nederkoorn PJ, van der Worp HB (2018). Restenosis and risk of stroke after stenting or endarterectomy for symptomatic carotid stenosis in the International Carotid Stenting Study (ICSS): secondary analysis of a randomised trial. *The Lancet Neurology*.

[106] Liistro F, Porto I, Grotti S, Ventoruzzo G, Vergallo R, Bellandi G (2012). Drug-eluting balloon angioplasty for carotid in-stent restenosis. *Journal of Endovascular Therapy*.

[107] Gandini R, Del Giudice C, Da Ros V, Sallustio F, Altobelli S, D’Onofrio A (2014). Long-term results of drug-eluting balloon angioplasty for treatment of refractory recurrent carotid in-stent restenosis. *Journal of Endovascular Therapy*.

[108] Piccoli G, Biondi-Zoccai G, Gavrilovic V, Radici V, Cancelli I, Frigatti P (2015). Drug-coated balloon dilation before carotid artery stenting of post-carotid endarterectomy restenosis. *Journal of Endovascular Therapy*.

[109] Tekieli Ł, Musiałek P, Kabłak-Ziembicka A, Trystuła M, Przewłocki T, Legutko J (2019). Severe, recurrent in-stent carotid restenosis: endovascular approach, risk factors. Results from a prospective academic registry of 2637 consecutive carotid artery stenting procedures (TARGET-CAS). *Advances in Interventional Cardiology*.

[110] Haupert G, Ammi M, Hersant J, Daligault M, Tesson P, Papon X (2020). Treatment of Carotid Restenoses after Endarterectomy: a Retrospective Monocentric Study. *Annals of Vascular Surgery*.

[111] Bamford J, Sandercock P, Dennis M, Burn J, Warlow C (1991). Classification and natural history of clinically identifiable subtypes of cerebral infarction. *Lancet*.

[112] Bogousslavsky J, Van Melle G, Regli F (1988). The Lausanne Stroke Registry: analysis of 1,000 consecutive patients with first stroke. *Stroke*.

[113] Coward LJ, McCabe DJH, Ederle J, Featherstone RL, Clifton A, Brown MM (2007). Long-term outcome after angioplasty and stenting for symptomatic vertebral artery stenosis compared with medical treatment in the Carotid and Vertebral Artery Transluminal Angioplasty Study (CAVATAS): a randomized trial. *Stroke*.

[114] Compter A, van der Worp HB, Schonewille WJ, Vos JA, Boiten J, Nederkoorn PJ (2015). Stenting versus medical treatment in patients with symptomatic vertebral artery stenosis: a randomised open-label phase 2 trial. *The Lancet Neurology*.

[115] Markus HS, Larsson SC, Kuker W, Schulz UG, Ford I, Rothwell PM (2017). Stenting for symptomatic vertebral artery stenosis: the Vertebral Artery Ischaemia Stenting Trial. *Neurology*.

[116] Wang Y, Ma Y, Gao P, Chen Y, Yang B, Jiao L (2018). First Report of Drug-Coated Balloon Angioplasty for Vertebral Artery Origin Stenosis. *JACC: Cardiovascular Interventions*.

[117] Gruber P, Berberat J, Kahles T, Anon J, Diepers M, Nedeltchev K (2020). Angioplasty Using Drug-Coated Balloons in Ostial Vertebral Artery Stenosis. *Annals of Vascular Surgery*.

[118] Wang Y, Feng Y, Wang T, Ma Y, Gao P, Chen J (2021). Drug-coated balloon for vertebral artery origin stenosis: a pilot study. *Journal of NeuroInterventional Surgery*.

[119] Wang M, Wang F, Liu Y, Yu L (2021). Comparison of Drug-Coated Balloons to Bare Metal Stents in the Treatment of Symptomatic Vertebral Artery-Origin Stenosis: a Prospective Randomized Trial. *World Neurosurgery*.

[120] Varcoe R, Smith W (2011). Use of a cutting balloon and a paclitaxel-coated balloon to treat recurrent subclavian in-stent restenosis causing coronary subclavian steal syndrome. *Cardiovascular Revascularization Medicine*.

[121] Silingardi R, Lauricella A, Cataldi V, Njila MKS, Coppi G (2014). Mechanical thrombectomy in proximal subclavian artery in-stent occlusion. *Cardiovascular Intervention and Therapeutics*.

[122] Albashaireh D, Khoueiry G, Mogabgab O, Saleh Q, Foster M, Abi Rafeh N (2017). Drug coated balloon angioplasty for subclavian artery stenosis: a potential novel indication. *Cardiovascular Revascularization Medicine*.

[123] Remonda L, Diepers M, Berberat J, Kahles T, Anon J, Nedeltchev K (2021). Drug-Coated Balloon Treatment in Symptomatic Intracranial High Grade Stenosis: A Retrospective Study of 33 Patients. *Clinical Neuroradiology*.

[124] Yang XM, Lu J, Qi P, Wang JJ, Hu S, Chen KP (2020). Preliminary application of drug-coated balloon in patients with symptomatic intracranial vertebrobasilar artery stenosis. *Chinese Journal of Surgery*.

[125] Peng G, Zhang Y, Miao Z (2020). Incidence and Risk Factors of in-Stent Restenosis for Symptomatic Intracranial Atherosclerotic Stenosis: a Systematic Review and Meta-Analysis. *AJNR. American Journal of Neuroradiology*.

[126] Mazighi M, Maurice JPS, Bresson D, Szatmary Z, Houdart E (2010). Platelet Aggregation in Intracranial Stents may Mimic in-Stent Restenosis. *AJNR. American Journal of Neuroradiology*.

[127] Alexander MJ, Zauner A, Chaloupka JC, Baxter B, Callison RC, Gupta R (2019). WEAVE Trial: Final Results in 152 On-Label Patients. *Stroke*.

[128] Alexander MJ, Zauner A, Gupta R, Alshekhlee A, Fraser JF, Toth G (2021). The WOVEN trial: Wingspan one-year Vascular Events and Neurologic Outcomes. *Journal of NeuroInterventional Surgery*.

[129] Cui R, Yan L, Kang K, Yang M, Yu Y, Mo D (2021). Long-Term Outcome of Enterprise Stenting for Symptomatic ICAS in a High-Volume Stroke Center. *Frontiers in Neurology*.

[130] Chatterjee AR, Derdeyn CP (2015). Stenting in Intracranial Stenosis: Current Controversies and Future Directions. *Current Atherosclerosis Reports*.

[131] Kurre W, Brassel F, Brüning R, Buhk J, Eckert B, Horner S (2012). Complication rates using balloon-expandable and self-expanding stents for the treatment of intracranial atherosclerotic stenoses: analysis of the INTRASTENT multicentric registry. *Neuroradiology*.

[132] Kurre W, Berkefeld J, Brassel F, Brüning R, Eckert B, Kamek S (2010). In-hospital complication rates after stent treatment of 388 symptomatic intracranial stenoses: results from the INTRASTENT multicentric registry. *Stroke*.

[133] Abou-Chebl A, Bashir Q, Yadav JS (2005). Drug-eluting stents for the treatment of intracranial atherosclerosis: initial experience and midterm angiographic follow-up. *Stroke*.

[134] Boulos AS, Agner C, Deshaies EM (2005). Preliminary evidence supporting the safety of drug-eluting stents in neurovascular disease. *Neurological Research*.

[135] Qureshi AI, Kirmani JF, Hussein HM, Harris-Lane P, Divani AA, Suri MFK (2006). Early and intermediate-term outcomes with drug-eluting stents in high-risk patients with symptomatic intracranial stenosis. *Neurosurgery*.

[136] Gupta R, Al-Ali F, Thomas AJ, Horowitz MB, Barrow T, Vora NA (2006). Safety, feasibility, and short-term follow-up of drug-eluting stent placement in the intracranial and extracranial circulation. *Stroke*.

[137] Steinfort B, Ng PP, Faulder K, Harrington T, Grinnell V, Sorby W (2007). Midterm outcomes of paclitaxel-eluting stents for the treatment of intracranial posterior circulation stenoses. *Journal of Neurosurgery*.

[138] Miao ZR, Feng L, Li S, Zhu F, Ji X, Jiao L (2009). Treatment of symptomatic middle cerebral artery stenosis with balloon-mounted stents: long-term follow-up at a single center. *Neurosurgery*.

[139] Natarajan SK, Ogilvy CS, Hopkins LN, Siddiqui AH, Levy EI (2010). Initial experience with an everolimus-eluting, second-generation drug-eluting stent for treatment of intracranial atherosclerosis. *Journal of Neurointerventional Surgery*.

[140] Fields JD, Petersen BD, Lutsep HL, Nesbit GM, Liu KC, Dogan A (2011). Drug eluting stents for symptomatic intracranial and vertebral artery stenosis. *Interventional Neuroradiology*.

[141] Vajda Z, Aguilar M, Göhringer T, Horváth-Rizea D, Bäzner H, Henkes H (2012). Treatment of intracranial atherosclerotic disease with a balloon-expandable paclitaxel eluting stent: procedural safety, efficacy and mid-term patency. *Clinical Neuroradiology*.

[142] Song L, Li J, Gu Y, Yu H, Chen B, Guo L (2012). Drug-eluting vs. bare metal stents for symptomatic vertebral artery stenosis. *Journal of Endovascular Therapy*.

[143] Lee J, Jo S, Jo K, Kim M, Lee S, You S (2013). Comparison of Drug-eluting Coronary Stents, Bare Coronary Stents and Self-expanding Stents in Angioplasty of Middle Cerebral Artery Stenoses. *Journal of Cerebrovascular and Endovascular Neurosurgery*.

[144] Park S, Lee D, Chung W, Lee DH, Suh DC (2013). Long-term Outcomes of Drug-eluting Stents in Symptomatic Intracranial Stenosis. *Neurointervention*.

[145] Kurre W, Aguilar-Pérez M, Fischer S, Arnold G, Schmid E, Bäzner H (2015). Solving the Issue of Restenosis After Stenting of Intracranial Stenoses: Experience with Two Thin-Strut Drug-Eluting Stents (DES)-Taxus Element™ and Resolute Integrity™. *Cardiovascular and Interventional Radiology*.

[146] Kim J, Ban SP, Kim YD, Kwon O (2020). Long-term outcomes of drug-eluting stent implantation in patients with symptomatic extra- and intracranial atherosclerotic stenoses. *Journal of Cerebrovascular and Endovascular Neurosurgery*.

[147] Jia B, Zhang X, Ma N, Mo D, Gao F, Sun X (2022). Comparison of Drug-Eluting Stent With Bare-Metal Stent in Patients With Symptomatic High-grade Intracranial Atherosclerotic Stenosis: A Randomized Clinical Trial. *JAMA Neurology*.

[148] Ye G, Yin X, Yang X, Wang J, Qi P, Lu J (2019). Efficacy and safety of drug-eluting stent for the intracranial atherosclerotic disease: a systematic review and meta-analysis. *Journal of Clinical Neuroscience*.

[149] Siontis GCM, Stefanini GG, Mavridis D, Siontis KC, Alfonso F, Pérez-Vizcayno MJ (2015). Percutaneous coronary interventional strategies for treatment of in-stent restenosis: a network meta-analysis. *The Lancet*.

[150] Alfonso F, Cuesta J (2015). Long-Term Results of Drug-Coated Balloons for Drug-Eluting in-Stent Restenosis. *JACC: Cardiovascular Interventions*.

[151] Jeger RV, Farah A, Ohlow M, Mangner N, Möbius-Winkler S, Leibundgut G (2018). Drug-coated balloons for small coronary artery disease (BASKET-SMALL 2): an open-label randomised non-inferiority trial. *Lancet*.

[152] Connors JJ, Wojak JC (1999). Percutaneous transluminal angioplasty for intracranial atherosclerotic lesions: evolution of technique and short-term results. *Journal of Neurosurgery*.

[153] Dumont TM, Kan P, Snyder KV, Hopkins LN, Siddiqui AH, Levy EI (2012). Revisiting angioplasty without stenting for symptomatic intracranial atherosclerotic stenosis after the stenting and aggressive medical management for preventing recurrent stroke in intracranial stenosis (SAMMPRIS) study. *Neurosurgery*.

[154] Dumont TM, Sonig A, Mokin M, Eller JL, Sorkin GC, Snyder KV (2016). Submaximal angioplasty for symptomatic intracranial atherosclerosis: a prospective Phase i study. *Journal of Neurosurgery*.

[155] Kleber FX, Schulz A, Waliszewski M, Hauschild T, Böhm M, Dietz U (2015). Local paclitaxel induces late lumen enlargement in coronary arteries after balloon angioplasty. *Clinical Research in Cardiology*.

[156] Ann SH, Balbir Singh G, Lim KH, Koo BK, Shin ES (2016). Anatomical and Physiological Changes after Paclitaxel-Coated Balloon for Atherosclerotic De Novo Coronary Lesions: Serial IVUS-VH and FFR Study. *PLoS ONE*.

[157] Scheller B, Gemeinhardt O, Kleber FX (2021). Late lumen enlargement after treatment of de-novo lesions with drug coated balloon catheters – Glagov effect or plaque regression. *International Journal of Cardiology*.

[158] Quan V, Huynh R, Seifert PA, Kuchela A, Chen W, Sütsch G (2005). Morphometric analysis of particulate debris extracted by four different embolic protection devices from coronary arteries, aortocoronary saphenous vein conduits, and carotid arteries. *The American Journal of Cardiology*.

[159] Rogers C, Huynh R, Seifert PA, Chevalier B, Schofer J, Edelman ER (2004). Embolic protection with filtering or occlusion balloons during saphenous vein graft stenting retrieves identical volumes and sizes of particulate debris. *Circulation*.

[160] Margolis J, McDonald J, Heuser R, Klinke P, Waksman R, Virmani R (2007). Systemic nanoparticle paclitaxel (nab-paclitaxel) for in-stent restenosis i (SNAPIST-i): a first-in-human safety and dose-finding study. *Clinical Cardiology*.

[161] Huang H, Wu L, Guo Y, Zhang Y, Zhao J, Yu Z (2021). Treatment of the Carotid In-stent Restenosis: A Systematic Review. *Frontiers in Neurology*.

[162] Tank VH, Ghosh R, Gupta V, Sheth N, Gordon S, He W (2016). Drug eluting stents versus bare metal stents for the treatment of extracranial vertebral artery disease: a meta-analysis. *Journal of Neurointerventional Surgery*.

[163] Langwieser N, Buyer D, Schuster T, Haller B, Laugwitz K, Ibrahim T (2014). Bare Metal vs. Drug-Eluting Stents for Extracranial Vertebral Artery Disease: a Meta-Analysis of Nonrandomized Comparative Studies. *Journal of Endovascular Therapy*.

[164] Ortega-Gutierrez S, Lopez GV, Edgell RC, Mendez AA, Dandapat S, Roa JA (2019). Second Generation Drug-Eluting Stents for Endovascular Treatment of Ostial Vertebral Artery Stenosis: a Single Center Experience. *Frontiers in Neurology*.

[165] Goodney PP, Nolan BW, Eldrup-Jorgensen J, Likosky DS, Cronenwett JL (2010). Restenosis after carotid endarterectomy in a multicenter regional registry. *Journal of Vascular Surgery*.

[166] Stilo F, Montelione N, Calandrelli R, Distefano M, Spinelli F, Di Lazzaro V (2020). The management of carotid restenosis: a comprehensive review. *Annals of Translational Medicine*.

